# From Molecular Classification to Hereditary Cancer Syndrome Identification in Endometrial Cancer

**DOI:** 10.3390/genes17070801

**Published:** 2026-07-14

**Authors:** Laura Libera, Ileana Carnevali, Sofia Facchi, Nora Sahnane, Antonio Travaglino, Stefano La Rosa, Maria Grazia Tibiletti

**Affiliations:** 1 Unit of Pathology, Ospedale di Circolo, ASST Sette Laghi, 21100 Varese, Italy; laura.libera@asst-settelaghi.it (L.L.); sofia.facchi@asst-settelaghi.it (S.F.); nora.sahnane@asst-settelaghi.it (N.S.); 2 Hereditary Cancer Research Center, University of Insubria, 21100 Varese, Italy; ileana.carnevali@asst-settelaghi.it; 3 SSD Specialized Laboratory of Medical Genetics, Cytogenetics and Molecular Genetics, ASST Sette Laghi, 21100 Varese, Italy; 4 Unit of Pathology, Department of Medicine and Technological Innovation, University of Insubria, 21100 Varese, Italy; antonio.travaglino@uninsubria.it (A.T.); stefano.larosa@uninsubria.it (S.L.R.)

**Keywords:** endometrial cancer, germline variants, *POLE*, MMR, MSI, molecular classification, genetic counselling

## Abstract

**Background:** Endometrial carcinoma (EC) is one of the most common gynaecological malignancies, with a steadily increasing incidence worldwide. In 2013, a novel molecular classification of ECs enabled patient stratification into four groups with important clinical management implications: (1) DNA Polymerase Epsilon (*POLE*)-mutated, (2) microsatellite instability-high (MSI-H/dMMR) hypermutated, (3) tumours with low somatic copy number alteration (low-SCNA), and (4) tumours with high SCNA and p53 deficiency. This molecular classification should also be used as a tool to identify hereditary cancer syndromes, as MSI-ECs can be associated with Lynch Syndrome (LS, ORPHA:144) while somatic *POLE* mutations may reflect polymerase proofreading-associated polyposis (PPAP syndrome, ORPHA:447877). **Methods:** From 2022 to 2024, molecular classification of ECs was routinely performed on 188 consecutive cases. The results were correlated with family history and constitutional genetic testing. **Results:** Somatic testing revealed that 45 ECs were dMMR and 23 carried *POLE* variants. Five *POLE*-mutated ECs also displayed concomitant MSI. For all 68 patients, cancer genetic counselling was proposed, but only 41 accepted, and of these, 26 patients were eligible for the genetic test. None of the *POLE* somatic variants were proven to be constitutive, whereas LS was diagnosed in seven patients with dMMR-EC; interestingly, two of these patients displayed a *POLE*-mutated EC. **Conclusions:** Our data strongly suggest that molecular classification of ECs is important to improve the identification of LS and highlight the relevance of investigating all molecular markers at once in order to identify overlaps between *POLE* mutations and MMR defects.

## 1. Introduction

Endometrial carcinoma (EC) is one of the most common gynaecological cancers with a steady increase in incidence worldwide, being the sixth most frequent cancer in women [1,2]. Although most women present at an early stage of the disease, and therefore with a favourable prognosis and a 5-year overall survival (OS) of 81%, some patients present with advanced disease, which has an OS of 17% and 15% for stages IVA and IVB, respectively [3,4].

In 2013, a new molecular classification integrated previous histopathological findings with data from the combination of somatic mutational burden and somatic copy number alterations, allowing patients to be stratified with important implications for clinical decisions. Therefore, ECs have been classified into four groups: (1) DNA Polymerase Epsilon (*POLE*)-ultra mutated, (2) microsatellite instability-high hypermutated (MSI), (3) tumours with low somatic copy number alteration (low-SCNA), and (4) tumours with high SCNA. The first group was associated with endometrioid endometrial cancer (EEC) histology and good prognosis, the second and third groups were associated with EEC subtypes and intermediate prognosis; however, the fourth group was mainly composed of high-grade EECs and non-endometrioid endometrial cancers (NEECs) and was associated with the worst prognosis [5]. Obesity and prolonged exposure to oestrogen have been shown to be epidemiologically important risk factors in the onset of EC. In the gynaecological cancer spectrum, and more specifically in the context of uterine cancer, approximately 90–95% of tumours are considered sporadic, whereas 5–10% of ECs have a hereditary nature [6]. Inherited conditions predisposing patients to EC predominantly include not only Lynch and Cowden syndromes, but also polymerase proofreading-associated polyposis (PPAP), *MUTYH*-associated polyposis, and Peutz-Jeghers syndrome [6]. The impact of germline pathogenetic variants (PVs) of cancer genes on genetic susceptibility to inherited EC is relevant not only for EC patients, but also for healthy family members that could benefit from both standard and tailored surveillance strategies to improve prevention.

The recent EC molecular classification includes markers such as *POLE* somatic mutations and MSI status and could reveal hereditary conditions. Indeed, it is well known that ECs with microsatellite instability could be suggestive of Lynch Syndrome (ORPHA 144), while a *POLE* mutation identified in tissues could have a germline origin, revealing PPAP syndrome (ORPHA447877). Therefore, the recent EC classification is an important tool to help identify genetic cancer syndromes and to subsequently activate cancer prevention in high-risk patients.

In this study, we will investigate the role of germline *POLE* and MMR genetic alterations in EC predisposition, discussing potential future applications and evaluating the benefit of performing routine genetic testing in order to adopt prevention and surveillance strategies in patients harbouring germline *POLE* or MMR pathogenic variants (PVs) and in unaffected family members.

## 2. Materials and Methods

### 2.1. Case Series

A total of 188 consecutive formalin-fixed and paraffin-embedded (FFPE) endometrial carcinomas (ECs) from 188 patients were collected from January 2022 to December 2024 at the Unit of Pathology of the University Hospital (ASST dei Sette Laghi) of Varese.

For each sample, a histopathological diagnosis was conducted according to the 5th edition of the World Health Organisation (WHO) Classification of Female Genital Tumours [7].

Table 1 lists the main clinical and histological characteristics of the EC series. In detail, the series included 167 ECs with endometrioid histotype, seven clear cell carcinomas, six serous carcinomas, six ECs with mixed histotype (two undifferentiated and endometrioid, two serous and clear cell, one endometrioid and serous, and one endometrioid and clear cell), one undifferentiated EC, and one carcinosarcoma. Overall, 150 cases were low-grade carcinomas (G1–G2), whereas 38 were high-grade tumours (G3). Molecular classification was performed, analysing all markers using an up-front strategy as described below. The tumour sample included both surgically removed ECs and biopsies, with a tumour content ranging from 10% to 70%. All cases were adequate for both molecular and immunohistochemical analyses.

### 2.2. Somatic POLE Exonuclease Domain Mutation Analysis

The mutational analysis of the exonuclease domain (Exonuclease Domain Mutation, EDM, exons 9, 10, 11, and 13) of *POLE* (NM_006231) was conducted using Next Generation Sequencing (NGS) and a Myriapod^®^ NGS Cancer panel DNA kit (NG033, Diatech Pharmacogenetics, Jesi, Italy). The NGS panel analyses the presence of single nucleotide variants (SNVs) in 17 oncogenes, namely *ALK*, *BRAF*, *EGFR*, *ERBB2*, *FGFR3*, *HRAS*, *IDH1*, *IDH2*, *KIT*, *KRAS*, *MET*, *NRAS*, *PDGFRA*, *PIK3CA*, *POLE*, *RET* and *ROS1*, by using a total of 100 ng of tumour DNA.

In detail, tumour DNA was extracted from three sections of 8 μm of FFPE ECs by using the Maxwell CSC automated system and Maxwell^®^ DNA FFPE kit (Promega, Madison, WI, USA) according to the manufacturer’s instructions. For each sample, a targeted amplicon-based library was constructed according to the manufacturer’s protocol. The libraries were diluted at 12pM and sequenced on Illumina MiSeq^®^ using a MiSeq Reagent Kit v2 Micro (300 cycles) (Illumina, San Diego, CA, USA).

The generated FASTQ were analysed by using Myriapod^®^ NGS Data Analysis version 5.0.7 according to the manufacturer’s protocol and mapped on the human reference genome GRCh37 (hg19). Sequencing data were filtered, ensuring a coverage of at least 500X and a variant allele frequency (VAF) higher than 5%. The identified genetic variants were divided into five classes according to the International Agency for Research on Cancer recommendations. *POLE* variants were classified with LOVD, Varsome databases [8,9] according to the American College of Medical Genetics—Association for Molecular Pathology criteria (ACMG-AMP) [10], and the *POLE* variants reported in Leon-Castillo et al. [11]. Class 4 and 5 variants were considered pathogenic, while class 1 and 2 variants were considered benign and not reported. Class 3 variants were considered of uncertain clinical significance (VUS).

### 2.3. Somatic Analysis for the Identification of MMR System Deficiency and p53 Alterations

To identify an MMR system deficiency (dMMR) or a p53 alteration, an immunohistochemical (IHC) analysis of MLH1, PMS2, MSH2, MSH6, and p53 proteins was performed on 3 μm FFPE tumour sections collected on Superfrost Plus slides. Tumour sections were processed automatically on the BenchMark ULTRA instrument with the OptiView DAB detection kit or Ultraview DAB detection kit (Ventana, Roche, Basilea, Switzerland) using the antibodies listed in Table 2.

For the interpretation of hMLH1, hMSH2, hMSH6, and hPMS2 protein IHC results, a case was considered negative for the expression of one of these proteins when no immunostaining was observed in the nuclei of tumour cells together with a normal positive staining in stromal cells, myocytes, normal epithelial cells, or lymphocytes (internal control). A sample was scored as dMMR when at least one MMR protein expression was negative. Generally, MLH1-PMS2 heterodimer loss is indicative of a *MLH1* gene defect, and MSH2-MSH6 heterodimer loss is indicative of a *MSH2* gene defect. The IHC loss of only MSH6 or PMS2 is indicative of a *MSH6* or *PMS2* gene defect, respectively [12].

The interpretation of p53 IHC results was conducted according to the criteria reported in Ronchi et al. [13]. In detail, the complete IHC loss (pattern null) and abnormal cytoplasmatic hyperexpression of p53 are indicative of a *TP53* gene defect.

### 2.4. Cancer Genetic Counselling and Germline Analysis of the MMR Gene, POLE, and POLD1

Cancer genetic counselling (CGC) was offered to all patients with ECs showing an MMR deficiency and/or a somatic variant of *POLE* in order to define the presence of a constitutive condition. CGC was also offered to patients with a suspected family history for a cancer predisposition syndrome independently from the somatic results.

A total of 41 patients were referred to the CGC service of the ASST Sette Laghi in Varese for assessment of their family’s cancer history and to discuss the possibility of germline genetic testing. All patients were evaluated according to the clinical criteria for the identification of Lynch Syndrome (Amsterdam and Bethesda-revised criteria [14]) and PPAP syndrome (attenuated polyposis [15]). According to ESGO guidelines [16], *MLH1* methylation analysis was performed on tumour DNA of MLH1-deficient samples to exclude LS by using the SALSA MS-MLPA kit ME011 (MRC Holland, Amsterdam, The Netherlands), as described in the manufacturer’s protocol.

The germline NGS analysis of *MLH1*, *MSH2*, *MSH6*, *PMS2*, *EPCAM*, *POLE,* and *POLD1* was performed by the Cogentech laboratory (Milan, Italy) using the custom OncoPan^®^ panel, which identifies SNVs, small insertions/deletions, and CNVs in the genes under investigation. Briefly, approximately 150–200 ng of DNA extracted from a blood sample was fragmented using the Sure Select Enzymatic Fragmentation Kit (Agilent Technologies Inc., Santa Clara, CA, USA). The NGS libraries were generated using Sure Select XT2 Low Input Custom Library Probes (Agilent Technologies Inc.) and sequenced on an Illumina MiSeq platform (Illumina Inc., San Diego, CA, USA) using a 2 × 150 bp paired-end sequencing protocol. Run quality was assessed using Illumina Sequencing Analysis Viewer v.1.9.1, whilst the bioinformatics pipeline for annotating variant calling files (VCFs) and copy number variations (CNVs) calling was developed by Cogentech (Milan, Italy), in collaboration with enGenome Software Company (Pavia, Italy). The paired-end reads were mapped to the human reference genome GRCh37 (hg19). The identified variants were filtered for a minimum coverage of more than 50 reads and a VAF greater than 10%. The identified variants were confirmed by Sanger sequencing using genomic DNA extracted from a second aliquot.

The identified genetic variants were divided into five classes according to the International Agency for Research on Cancer recommendations and were classified in accordance with the guidelines from Insight Classification (InSiGHT Variant Interpretation Committee: Mismatch repair Gene Variant Classification Criteria, 2018) [17]. *POLE* and *POLD1* variants were classified according to the LOVD database [8,18]. Class 4 and 5 variants were considered pathogenic, while class 1 and 2 variants were considered benign and not reported. Class 3 variants were considered of uncertain clinical significance (VUS).

## 3. Results

### 3.1. Somatic Molecular Profiling of Endometrial Carcinomas

Somatic molecular profiling was conducted on 188 consecutive ECs collected from 2022 to 2024 by the Anatomic Pathology Unit of ASST dei Sette Laghi of Varese. Performed analyses included NGS analysis to identify variants in the exonuclease domain (EDM) of *POLE*, and immunohistochemical analysis of the mismatch repair system (MMR proteins) and p53 in accordance with the molecular classification of endometrial carcinomas [7].

**Figure 1:**
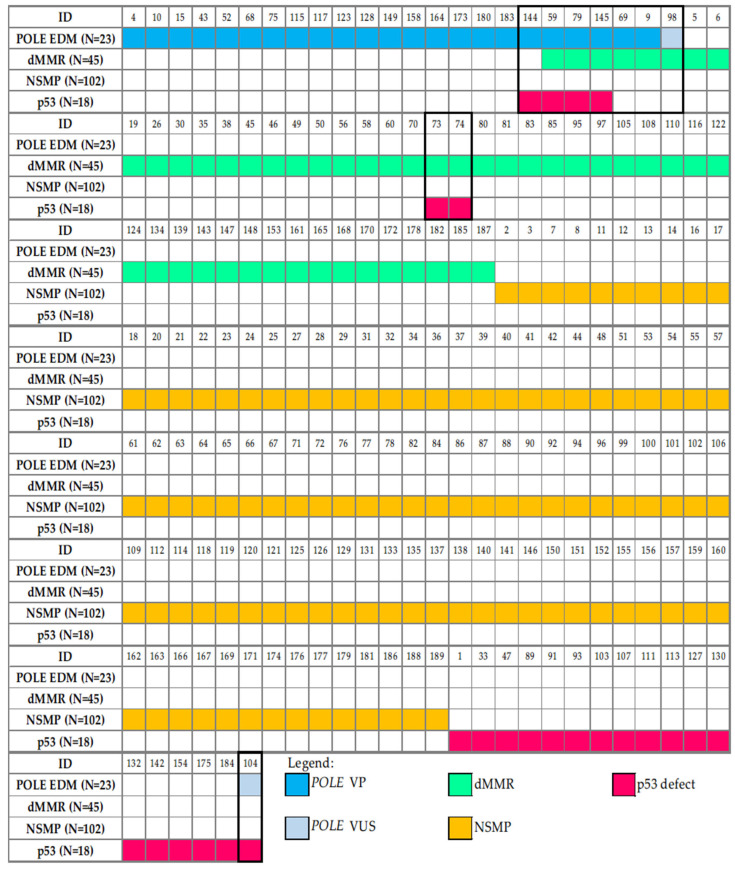
Molecular classification of 188 endometrial carcinomas. A total of 23 POLE-mutated ECs (blue), 45 dMMR ECs (green), 102 NSMPs (yellow), and 18 p53-deficient ECs (pink) were identified. The results of the molecular classification of the 188 ECs show a partial phenotypic overlap between molecular subgroups (samples highlighted by black boxes).

The NGS analysis of *POLE* EDM (exons 9, 10, 11, and 13) identified a total of 23 ECs exhibiting at least one *POLE* that was likely pathogenic or a pathogenic variant (class 4 and 5, blue in Figure 1), and two ECs with a *POLE* variant of uncertain significance (VUS, class 3, light blue in Figure 1), with a VAF ranging from 5% to 66%. Table 3 lists the *POLE* EDM variants identified.

IHC analysis of MMR system proteins identified an MMR defect (dMMR) in 50 ECs (green in Figure 1). In detail, 33 carcinomas were negative for the MLH1-PMS2 heterodimer, nine cases were MSH2-MSH6-negative, four cases exhibited a deficiency in MSH6 alone, one case was negative for PMS2 alone (ID116), one case showed an MLH1-PMS2 defect together with MSH6 loss (ID143), one case was MSH2-MSH6- and PMS2-negative (ID147), and one case was negative for MLH1 alone (ID178).

Abnormal p53 expression was observed in 24 ECs, including cases lacking protein expression (null pattern) and cases with overexpression of the mutated p53 protein, which tends to accumulate in tumour cells (pink in Figure 1). Interestingly, among the 24 p53-defective ECs, only five carcinomas had a serous histotype, whereas the remaining cases had an endometrioid (nine cases), mixed (five cases), clear cell (four cases), or carcinosarcoma (one case) histotype.

According to the ESGO/ESTRO/ESP guidelines for the management of patients with endometrial cancer [19,20], *POLE* mutation is the driving classifier criterium, followed by dMMR. Consequently, the EC cohort was classified as follows (Figure 1): 23 *POLE*-mutated ECs (blue), which also included six multi-classified cases showing MMR and/or p53 defects together with *POLE* mutation; 45 dMMR ECs (green), including three multi-classified ECs; 102 ECs without a specific molecular profile (NSMP, yellow); and 18 p53-deficient ECs (pink).

### 3.2. Multi-Classified ECs

It is interesting to note that there is a partial overlap between molecular subgroups, which are not mutually exclusive (Figure 1, Table 4). Indeed, five out of 23 *POLE*-mutated ECs (ID 59, 79, 145, 69, and 9) also exhibited a defect in the MMR system, and three of the 23 revealed p53 alterations (ID59, 79, 145). The MMR defect in these five cases was also confirmed by MSI fragment analysis assay according to Libera et al. [21]. Moreover, the two MLH1-PMS2 defective cases revealed *MLH1* promoter hypermethylation (ID69 and 79). The remaining three cases (ID9, 145, and 59) were MSH6 defective. ID144 EC showed the concomitance of *POLE* mutation and p53 defection.

Similarly, among the 45 ECs classified in the dMMR group, two ECs also showed abnormal expression of p53 (ID73 and 74), and ID98 revealed a concomitant *POLE* VUS. Finally, ID104 EC was characterised by a p53 defect and a *POLE* VUS.

These 10 cases, marked by a black box in Figure 1, are termed ‘multiple-classifiers’, as previously described by Vermij et al. and De Vitis et al. [19,22]. Table 4 describes in detail the histological and molecular features of the 10 ‘multi-classifier’ cases. In agreement with ESGO/ESTRO/ESP recommendations, multi-classifier ECs which were *POLE*-mutated and dMMR and *POLE*-mutated with p53 deficiency were all classified as *POLE*-mutated ECs.

### 3.3. Cancer Genetic Counselling and Germline Results

Cancer genetic counselling (CGC) was offered to patients with dMMR ECs or somatic *POLE*-mutated ECs in order to identify Lynch Syndrome and PPAP patients. Among these, 41 out of 68 patients (compliance of 60%) referred to the CGC service, where family cancer history, histological reports, and molecular reports were also collected. A total of 12 patients showed a somatic *POLE* pathogenetic variant (five of them together with dMMR), while 29 patients had dMMR ECs (18 dMLH1-PMS2, seven dMSH2-MSH6, one dMLH1, one dMSH6, one dMSH2-MSH6-PMS2, and one dPMS2).

*MLH1* methylation analysis was requested during CGC and was performed on the 19 dMLH1 ECs according to ESGO guidelines and the AIFET position paper [16,23]; 15 cases resulted in *MLH1* hypermethylation and were excluded from further germline analysis. Thereby, germline analysis for Lynch Syndrome and/or PPAP was offered to a total of 26 patients (12 patients with POLE-mutated ECs and 14 dMMR ECs).

Targeted analysis of *POLE* variants was performed in a germline setting for all patients with *POLE*-mutated ECs. All patients with dMMR ECs were also tested for the MMR genes that were deficient in IHC analysis. If the family history was suggestive for a polyposis syndrome, the germline analysis was also extended to the *POLD1* gene.

The germline analysis performed on 26 patients identified seven patients with a germline variant in an MMR gene (26.9%) and one patient with a class 3 variant of the *POLD1* gene (ID69). One patient showed a class 4 *MSH2* variant (ID80), four patients exhibited a class 5 variant of the *MSH6* gene (ID59, 145, 58, and 98), one patient had a class 5 *MSH2* variant, and one patient was a carrier of a class 5 *PMS2* variant. Somatic and germline molecular results in relationship with family histories and EC histological types are reported in Table 5. Notably, two patients with *POLE*-mutated ECs showed the presence of constitutive *MSH6* variants.

## 4. Discussion

Given the large body of literature on the management of endometrial carcinoma, in 2020 the European Society of Gynaecology Oncology (ESGO), the European Society for Radiotherapy and Oncology (ESTRO), and the European Society of Pathology (ESP) decided to update evidence-based guidelines in order to complement the ESMO clinical practice previously published [24] and to improve the quality of care for women with endometrial carcinomas across Europe and worldwide [20]. These guidelines outlined the relevance of conventional pathologic analysis but encouraged molecular classification in all endometrial carcinomas, especially high-grade tumours. Molecular classification includes *POLE* mutation analysis, as well as MMR and identification of p53 deficiency. From 2022, molecular classification of ECs was routinely performed in our centre. In agreement with other data reported in the literature [25], our data revealed that about 12% of ECs were classified as *POLE*-mutated, 24% as dMMR-hypermutated, 54% as NSMP (low-SCNA), and 10% as p53-deficient (high-SCNA). Interestingly, a subset of ECs (10 cases) were classed as “multiple-classifiers”, showing concurrent molecular markers: *POLE* PV EDM in co-occurrence with MMR (five cases; Figure 1) or p53 deficiency (one case), MMR defects and p53 abnormalities (two cases), and *POLE* VUS EDM in concomitance with dMMR (one case) or p53 deficiency (one case).

The co-occurrence of a germline *POLE* variant with a somatic MMR-defective phenotype was recently reported in colorectal cancer [26], demonstrating a close interaction between the two DNA repair systems.

Approximately 3% of all ECs and about 10% of mismatch repair-deficient (dMMR) ECs are causally related to germline pathogenetic variants of one MMR gene [25]. Testing for MMR status in EC patients has been shown to be relevant, including pre-screening to identify women with Lynch Syndrome at high risk of gynaecological and colorectal cancers [16,23]. To identify patients with Lynch Syndrome, all MMR-defective ECs that test negative for somatic *MLH1* methylation should be investigated for MMR germline variants.

Increased lifetime risk of EC has also been observed in carriers of *POLE/POLD1* germline pathogenetic variants. These genes code for DNA polymerase epsilon and delta, which are involved in DNA replication and repair, and their pathogenetic variants are responsible for PPAP cancer syndrome (ORPHA447877), an autosomal dominant syndrome associated with attenuated polyposis, colorectal cancer, EC, brain tumours and, probably, ovarian cancer [27]. In order to investigate the presence of PPAP syndrome, CGC should also be offered to all patients with *POLE*-mutated ECs.

On these bases, the molecular classification of ECs is important not only to improve the quality of care for women with ECs but also to diagnose hereditary cancer syndromes, with important implications for patient surveillance and the genetic counselling of their relatives. In fact, in this study, Cancer Genetic Counselling (CGC) was offered to a total of 23 patients with ECs characterised by somatic *POLE* mutations and 45 patients with dMMR ECs, taking advantage of the molecular classification of their tumours. Twelve out of 23 patients with somatic *POLE* variants and 29 out of 45 patients with dMMR ECs consented to CGC (compliance 60%).

For patients with both *POLE* somatic variants and dMMR ECs, a germline genetic test for *POLE* and MMR genes was performed and, surprisingly, two *MSH6* germline pathogenetic variants were identified (Table 5): *MSH6* c.3775_3776del and *MSH6* c.2883_2884insT. No *POLE* germline variants were found in women with *POLE*-mutated ECs.

In the subset of dMMR ECs, 14 patients were tested for Lynch Syndrome, and an MMR pathogenetic variant was identified in five patients: two *MSH6*, two *MSH2,* and one *PMS2* (see Table 5). Interestingly, four EC patients who were carriers of germline pathogenetic variants of *MSH6* (cases 59, 145, 98) and *PMS2* (case 143) were negative for Amsterdam criteria, showing negative family history for colorectal and LS-related cancers. It is noteworthy that these four patients, who were carriers of *MSH6* or *PMS2* pathogenic variants, would have been missed if clinical criteria alone were taken into account. In summary, in this study, seven out of 188 (3.7%) patients with Lynch Syndrome were identified and no PPAP syndrome was diagnosed. The incidence of LS in this study is in agreement with data from the literature and, in addition, the *MSH6* gene is confirmed as the most relevant gene in Lynch-Syndrome-related ECs (4/188, 2.1%), as described by other authors [28]. Nevertheless, LS incidence in this cohort could be underestimated due to the intrinsic limitations of our testing approach (germline testing was proposed only to women with somatic-altered tumours) and to the low rate of women who attended CGC (60%).

**Figure 2:**
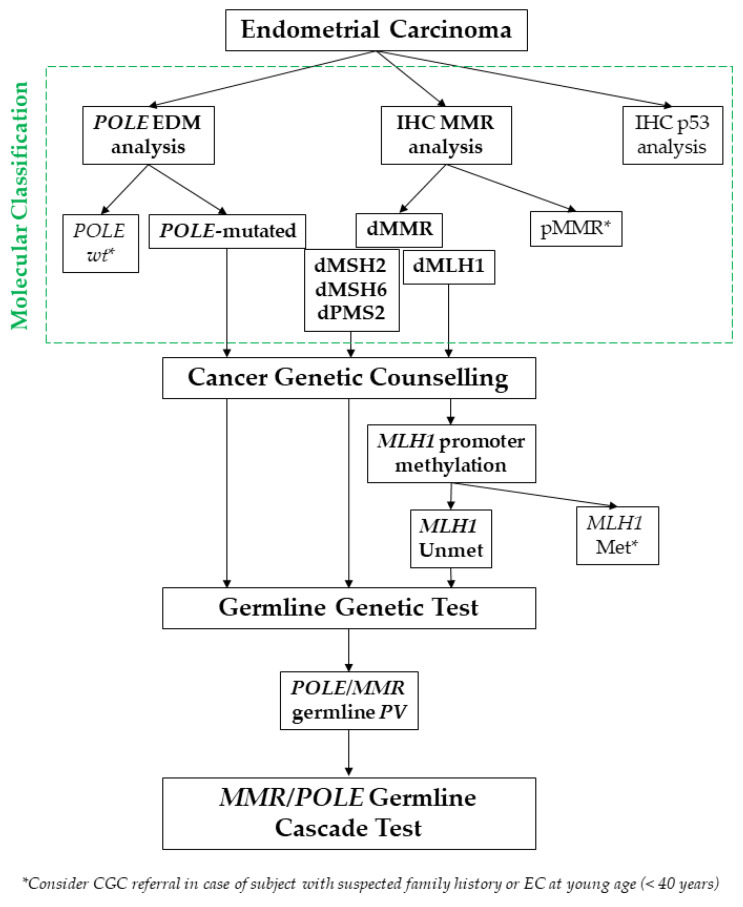
Flowchart for the identification of cancer genetic syndromes by using EC molecular classification.

Our data strongly suggest that EC molecular classification is useful to improve LS identification and outlines the importance of assessing all EC molecular classification markers at once, avoiding a reflex approach. In fact, in our series, the co-occurrence of *POLE* PV mutations and MMR defects was identified in five ECs, and two of these patients revealed LS, which could be misdiagnosed using a reflex approach. Our data supports the implementation of a new flowchart (Figure 2), with the aim of identifying genetic cancer syndromes in EC patients and their high-risk relatives, starting from EC molecular classification.

This is the first report that exploited EC molecular classification in order to identify hereditary cancer syndromes, and outlined the relevance of up-front marker testing to avoid underestimation of cancer syndromes.

## 5. Conclusions

The molecular classification of ECs is important not only to improve the quality of care for women with ECs but also to diagnose hereditary cancer syndromes, with important implications for their surveillance and the genetic counselling of their high-risk family members. Molecular classification should include all molecular markers up front, avoiding a reflex approach, in order to identify ‘multiple-classifier’ cases. Germline genetic testing offered to EC patients with somatic MMR and *POLE* mutations is relevant to identify LS families lacking clinical LS criteria.

## Figures and Tables

**Table 1:** Clinical and histopathological features of the series.

Histopathological Features	188 EC (%)
**Mean Age at Onset** (Range)	**66 years** (37–89)
**Histotype**	
Endometrioid	167 (88.8%)
Clear cell	7 (3.8%)
Serous	6 (3.2%)
Mixed	6 (3.2%)
Undifferentiated	1 (0.5%)
Carcinosarcoma	1 (0.5%)
**TNM Stage**	
pT1a-1b	157 (83.5%)
pT2-3	23 (12.2%)
pT not available	8 (4.3%)
pN0	138 (73.4%)
pN1-2	9 (4.8%)
pN not available	41 (21.8%)
**Grade**	
G1-G2	150 (79.8%)
G3	38 (20.2%)

**Table 2:** Immunohistochemical monoclonal antibody list.

Antibody	Clone	Dilution	Distribution
MLH1	M1	Pure	Ventana, Roche, Switzerland
MSH2	G219-1129	Pure	Ventana, Roche, Switzerland
MSH6	44	Pure	Ventana, Roche, Switzerland
PMS2	EPR3947	Pure	Cell Marque, Merck, Rahway, NJ, USA
p53	Bp53-11	Pure	Ventana, Roche, Switzerland

**Table 3:** List of *POLE* somatic variants identified by NGS analysis.

ID	Coding Variant	Protein Variant	VAF (%)	Variant Class
4	c.1231G>C	p.(Val411Leu)	66%	5
9	c.1387G>C	p.(Ala463Pro)	9%	4
10	c.1231G>C	p.(Val411Leu)	15%	5
15	c.1366C>T	p.(Ala456Pro)	33%	5
43	c.1100T>C	p.(Phe367Ser)	23%	5
52	c.857C>G	p.(Pro286Arg)	36%	5
59	c.1372T>C	p.(Tyr458His)	32%	4
68	c.1231G>C	p.(Val411Leu)	5%	5
69	c.890C>T	p.(Ser297Phe)	30%	4
75	c.1231G>C	p.(Val411Leu)	18%	5
79	c.1331T>A	p.(Met444Lys)	15%	5
115	c.857C>G	p.(Pro286Arg)	11%	5
117	c.857C>G	p.(Pro286Arg)	13%	5
123	c.1231G>C	p.(Val411Leu)	25%	5
128	c.1376C>T	p.(Ser459Phe)	40%	5
144	c.857C>G	p.(Pro286Arg)	44%	5
145	c.1376C>T	p.(Ser459Phe)	45%	5
149	c.890C>A	p.(Ser297Tyr)	17%	4
158	c.1366C>T	p.(Ala456Pro)	22%	5
164	c.857C>G	p.(Pro286Arg)	36%	5
173	c.1376C>A	p.(Ser459Tyr)	26%	5
180	c.1231G>C	p.(Val411Leu)	26%	5
183	c.1231G>C	p.(Val411Leu)	26%	5
98	c.1496_1497delinsTG	p.(Thr499Met)	15%	3
104	c.1074C>G	p.(Ile358Met)	16%	3

Legend: ID, sample identification number; VAF, variant allele frequency; Variant class 3, variant of unknown significance (VUS); Class 4, likely pathogenic variant; Class 5, pathogenic variant.

**Table 4:** Histological and molecular features of the ‘multi-classifier’ cases.

ID	Molecular Class	Histology	POLE	MSH2	MSH6	MLH1	PMS2	p53
9	1	Endometrioid						
69	1	Endometrioid						
79	1	Endometrioid						
145	1	Endometrioid						
59	1	Mixed						
144	1	Endometrioid						
73	2	Endometrioid						
74	2	Endometrioid						
98	2	Endometrioid						
104	4	Mixed						

Legend: ID, sample identification number; Molecular class 1, POLE-mutated EC; Class 2, dMMR-ECs; Class 4, p53-defective copy-number high ECs; Blue, presence of POLE pathogenetic variant; Light Blue, presence of POLE VUS variant; Green, loss of MMR expression; Pink, p53 deficiency; White, no defect.

**Table 5:** Somatic and germline molecular results in relationship with family history and EC histological types.

	Somatic Analysis								Germline Results			
ID	Molecular Class	Histology	POLE	MSH2	MSH6	MLH1	PMS2	p53	Amsterdam Criteria	Germline Results	HGVS Variant Nomenclature	Variant Class
4	1	E	**p.(Val411Leu)**						No	wt		
10	1	E	**p.(Val411Leu)**						No	wt		
43	1	E	**p.(Phe367Ser)**						No	wt		
123	1	E	**p.(Val411Leu)**						**YES**	wt		
164	1	E	**p.(Pro286Arg)**						**YES**	wt		
180	1	E	**p.(Val411Leu)**						No	wt		
183	1	E	**p.(Val411Leu)**						**YES**	wt		
9	1	E	**p.(Ala463Pro)**		**A**				No	wt		
79	1	E	**p.(Met444Lys)**			**A**	**A**	**A**	**YES**	wt		
69	1	E	**p.(Ser297Phe)**			**A**	**A**		No	***POLD1***	**NM_002691.4(POLD1):c.427G>A p.(Gly143Ser)**	**3**
59	1	M	**p.(Tyr458His)**		**A**			**A**	No	***MSH6***	NM_000179.3(MSH6):c.2883_2884insT p.(Ile962Tyrfs*4)	**5**
145	1	E	**p.(Ser459Phe)**		**A**			**A**	No	***MSH6***	NM_000179.3(MSH6):c.3775_3776del p.(Asn1259Cysfs*15)	**5**
19	2	E	wt			**A**	**A**		No	wt		
134	2	E	wt			**A**	**A**		**YES**	wt		
187	2	E	wt			**A**	**A**		**YES**	wt		
116	2	E	wt				**A**		**YES**	wt		
147	2	E	wt	**A**	**A**		**A**		**YES**	wt		
60	2	E	wt	**A**	**A**				No	wt		
124	2	E	wt	**A**	**A**				No	wt		
178	2	E	wt			**A**			**YES**	wt		
122	2	E	wt	**A**	**A**				**YES**	***MSH2***	***MSH2*** **GRCh37 (hg19) NC_000002.12:g.(?_47630206)_(47643569_47656880)del p.(?)**	**5**
80	2	E	wt	**A**	**A**				**YES**	***MSH2***	**NM_000251.3(MSH2):c.792+2T>G p.(?)**	**4**
58	2	CC	wt	**A**	**A**				**YES**	***MSH6***	NM_000179.3(MSH6):c.*23_*26dup p.(?)	**5**
98	2	E	**p.Thr499Met** **(VUS)**		**A**				No	***MSH6***	NM_000179.3(MSH6):c.2764C>T p.(Arg922*)	**5**
143	2	E	wt		**A**	**A**	**A**		No	***PMS2***	**NM_000535.7(PMS2):c.137G>T p.(Ser46Ile)**	**5**

Legend: ID, sample identification number; Molecular class 1, POLE-mutated EC; Molecular class 2, dMMR-hypermutated EC; E, endometrioid; M, mixed; CC, clear cell; A, absence of MMR expression/p53 deficiency; White, no defect; Class, variant class; 3, VUS; 4, likely pathogenetic; 5, pathogenetic. Positive family history was considered if one first-degree relative of an EC patient was affected by endometrial, colorectal, or other LS-associated cancers according to Amsterdam criteria.

## Data Availability

The original contributions presented in this study are included in the article. Further inquiries can be directed to the corresponding author.

## Abbreviations

The following abbreviations are used in this manuscript:

ACMG-AMP	American College of Medical Genetics—Association for Molecular Pathology
ASST	Azienda Socio Sanitaria Territoriale
CGC	Cancer Genetic Counselling
EC	Endometrial Cancer
EEC	Endometrioid Endometrial Cancer
ESGO	European Society of Gynaecology Oncology
ESMO	European Society for Medical Oncology
ESP	European Society of Pathology
ESTRO	European Society for Radiotherapy and Oncology
IHC	Immunohistochemistry
LS	Lynch Syndrome
MMR	Mismatch Repair System
MSI	Microsatellite Instability
NEEC	Not Endometrioid Endometrial Cancer
NGS	Next-Generation Sequencing
NSMP	Non-Specific Molecular Profile
OS	Overall Survival
*POLE*	DNA Polymerase Epsilon
*POLE* EDM	POLE Exonuclease Domain Mutation
PPAP	Polymerase Proofreading-Associated Polyposis
PV	Pathogenic Variant
SCNA	Somatic Copy Number Alteration
VUS	Variant of Uncertain Significance
WHO	World Health Organisation
